# N,N-dimethyltryptamine compound found in the hallucinogenic tea ayahuasca, regulates adult neurogenesis in vitro and in vivo

**DOI:** 10.1038/s41398-020-01011-0

**Published:** 2020-09-28

**Authors:** Jose A. Morales-Garcia, Javier Calleja-Conde, Jose A. Lopez-Moreno, Sandra Alonso-Gil, Marina Sanz-SanCristobal, Jordi Riba, Ana Perez-Castillo

**Affiliations:** 1 Institute for Biomedical Research “A. Sols” (CSIC-UAM). Arturo Duperier 4, 28029 Madrid, Spain; 2 Spanish Center for Networked Biomedical Research on Neurodegenerative Diseases (CIBERNED), c/ Valderrebollo 5, 28031 Madrid, Spain; 3 Department of Cellular Biology, School of Medicine, Complutense University of Madrid, Plaza Ramón y Cajal, 28040 Madrid, Spain; 4 Cellular Neurobiology Laboratory, Neurobiology Department, UCS-UCM, Hospital Universitario Ramón y Cajal, IRYCIS, Madrid, Spain; 5 Department of Psychobiology and Behavioural Sciences Methods, Faculty of Psychology, Complutense University of Madrid, Carretera de Humera, 28223 Madrid, Spain; 6 Department of Neuropsychology and Psychopharmacology, Faculty of Psychology and Neuroscience, Maastricht University, Maastricht, 6229 ER The Netherlands

**Keywords:** Neuroscience, Drug discovery

## Abstract

N,N-dimethyltryptamine (DMT) is a component of the ayahuasca brew traditionally used for ritual and therapeutic purposes across several South American countries. Here, we have examined, in vitro and vivo, the potential neurogenic effect of DMT. Our results demonstrate that DMT administration activates the main adult neurogenic niche, the subgranular zone of the dentate gyrus of the hippocampus, promoting newly generated neurons in the granular zone. Moreover, these mice performed better, compared to control non-treated animals, in memory tests, which suggest a functional relevance for the DMT-induced new production of neurons in the hippocampus. Interestingly, the neurogenic effect of DMT appears to involve signaling via sigma-1 receptor (S1R) activation since S1R antagonist blocked the neurogenic effect. Taken together, our results demonstrate that DMT treatment activates the subgranular neurogenic niche regulating the proliferation of neural stem cells, the migration of neuroblasts, and promoting the generation of new neurons in the hippocampus, therefore enhancing adult neurogenesis and improving spatial learning and memory tasks.

## Introduction

N,N-dimethyltryptamine (DMT) is a natural compound found in numerous plant species and botanical preparations, such as the hallucinogenic infusion known as ayahuasca^1^ classified as a hallucinogenic compound that induces intense modifications in perception, emotion, and cognition in humans^2–4^. DMT is present in several animal tissues, such as the lung^5^ and brain^6^, being considered as an endogenous trace neurotransmitter with different physiological roles, including neural signaling and brain/peripheral immunological actions^7–10^. DMT is also present in human blood, urine, and cerebrospinal fluid^11–13^. Furthermore, some evidence suggests that DMT can be sequestered into and stored in the vesicle system of the brain and that environmental stress increases its levels in mammals’ central nervous system (CNS)^14–16^. DMT binds and exerts an agonist activity on subtypes 1A and 2A of the serotonin receptor (5-HT)^17,18^. These receptors are G-protein-coupled receptors (GPCRs) belonging to the family of serotonergic receptors and are involved in numerous cascades of intracellular signaling, with high expression in several regions of the CNS. Some studies have demonstrated that DMT also binds with low affinity to non-serotonergic receptors, such as the sigma-1 receptor (S1R). The S1R, traditionally thought to be an opioid receptor, is now classified as a highly conserved transmembrane protein member of an orphan family and located mainly in the membrane of the endoplasmic reticulum. σR‐1 is widespread in the CNS, mainly in the prefrontal cortex, hippocampus, and striatum^19^. Interestingly, in mammals, one of the natural endogenous ligands of the σR-1 is DMT^14^. This receptor has been associated with several cellular functions, including the brain, such as lipid transport, metabolism regulation, cellular differentiation, signaling (in response to stress), cellular protection against oxidant agents, myelination and, most recently, neurogenesis^20–24^.

Neurogenesis is the process of generating new functional neurons, mainly in the SVZ and the subgranular zone of the DG of the hippocampus. In mammals, this process occurs mostly during the prenatal period, being significantly reduced in adults^25–30^. In humans, although the presence of adult neurogenesis has been recently reported during aging^31–33^, most of the studies indicate that there are no substantial evidence to support it. A recent review by Duque and Spector suggests that, in adult age, preservation of the existing neurons is more important in contrast to the generation of new ones^34^. Neurogenesis is a complex process involving multiple cellular activities including the proliferation of neural stem cells (NSC; progenitors), migration and differentiation, survival, acquisition of cell fate and maturation, and integration of these newly born neurons in existing neuronal circuits. All these processes are precisely regulated by multiple factors^35^. Advancements in the knowledge of these factors and their mechanism of action could help us to investigate possible new instruments that will allow us to expand the limited endogenous neurogenic capacity of the adult brain and, consequently, opening new fields for the development of effective therapies in the treatment of brain damage and neurodegenerative diseases.

Neurodegenerative diseases (including Parkinson’s, Alzheimer’s, Hungtinton’s, etc.) and acute neural damage (such as stroke and traumatic brain injury) are characterized by a gradual and selective loss of neurons in the affected regions of the nervous system. One common feature in these disorders is an impairment in the proliferation of progenitor cells in the neurogenic niches^36,37^. In animal models reproducing the pathological hallmarks of Alzheimer disease, a loss of neurogenic capacity has been described in the SVZ^38^. This decrease is also observed in the postmortem brains of Parkinson’s patients, suggesting that the loss of neurogenic activity is due to the loss of dopamine, affecting the neural precursors in the adult^39^. These data support the fact that in neurodegenerative diseases, such as Alzheimer’s and Parkinson’s, not only degeneration and death of mature neurons occur but also the process of formation of new neuronal progenitors in the adult brain is negatively affected. According to these data, the stimulation of endogenous populations of stem cells and neuronal progenitors could be a promising approach to improve the functionality of some of the regions affected by neurodegenerative pathologies. In fact, the stimulation of neurogenesis has already been proposed as a new therapeutic strategy for psychiatric and neurological diseases^40–44^, and several studies have reported that the clinical efficacy of antidepressant drugs is frequently linked to the capacity of these drugs to induce neurogenesis^45–48^.

Based on the data above mentioned including our results on the potent neurogenic effect of the other components of the Ayahuasca^49^, the main objective of this work was to analyze the possible role of DMT in adult neurogenesis, as well as to elucidate its mechanism of action.

## Materials and methods

### Animals and ethics

Adult male C57/BL6 mice (3-months old) were used in this study following the animal experimental procedures and protocols specifically approved by the “Ethics Committee for Animal Experimentation” of the Institute for Biomedical Research (CSIC-UAM) and carried out in accordance with the European Communities Council, directive 2010/63/EEC and National regulations, normative RD1386/2018. Adequate measures were taken to minimize the pain or discomfort experienced by the mice.

### Adult precursor isolation

NSCs were isolated from the subgranular zone (SGZ) of the hippocampus of adult mice and prepared the following previously described methods^49^. A total number of 24 animals were used divided into four different pools (six animals/pool). Briefly, tissue was carefully dissected, dissociated in DMEM medium with glutamine, gentamicin, and fungizone, and then digested with 0.1% trypsin-EDTA + 0.1% DNAse + 0.01% hyaluronidase for 15 min at 37 °C. The isolated stem cells were seeded into six-well dishes at a density of ~40,000 cells per cm^2^ in DMEM/F12 (1:1) containing 10 ng/mL epidermal growth factor (EGF), 10 ng/mL fibroblast growth factor (FGF), and N2 medium.

### Neurosphere culture and treatments

After 1 week in culture under standard conditions, small neural progenitor-enriched growing spheres known as neurospheres (NS) were formed. At this point, with all NS having the same stage and size, cultures were treated daily for 7 days under proliferative conditions (in the presence of exogenous growth factors, EGF and FGF) with vehicle or DMT (1 μM). Some cultures were individually pre-treated for 1 h at 1 μM with antagonists of the different receptors that bind DMT: BD1063 (BD, sigma-1R), methiothepin (Met, 5-HT1A/2A), ritanserin (Rit, 5-HT2A), and WAY100635 (WAY, 5-HT1A). None of the tested drugs affected the viability of cultured cells at this dosage (data not shown).

### Growth and proliferation measurements

After 7 days on culture in the presence or not of compounds, proliferation and growth analysis was assessed, and the number and size of NS in ten wells per condition were quantified using the Nikon Digital Sight, SD-L1 software. At least 50 neurospheres per condition were quantified. Next, some of these proliferating NS were used for immunoblotting analysis, while others were seeded onto coverslips and cultured again in the presence of DMT (1 μM) and/or DMT + antagonist (1 μM) during 24 h under differentiation conditions (medium containing 1% fetal bovine serum and without exogenous growth factors). NS were then fixed in paraformaldehyde 4% during no more than 20 min, and then immunocytochemistry analysis was performed using a specific antibody for proliferation. The remaining NS were cultured for differentiation studies.

### Differentiation of NS cultures

To determine the ability of DMT to stimulate neurons, astrocytes, or oligodendrocytes formation, NS from 7-day-old cultures were seeded onto poly-l-lysine-precoated six-well plates and/or on coverslips and cultured in the presence of DMT (1 μM) and/or DMT + antagonist (1 μM) under differentiation conditions (medium containing 1% fetal bovine serum and without exogenous growth factors). Once neurospheres were differentiated (72 h), those grown on coated six-well plates were used for immunoblotting and those on coverslips for immunocytochemical analysis.

### Protein extraction and western blot analysis

Cultured NS on coated six-well plates were resuspended in ice-cold cell lysis buffer (Cell Signaling Technology) with protease inhibitor cocktail (Roche) and incubated for 15–30 min on ice. A total amount of 30 µg of protein was loaded on a 10% or 12% SDS-PAGE gel and transferred to nitrocellulose membranes (Protran, Whatman). The membranes were blocked in Tris-buffered saline with 0.05% Tween-20 and 5% skimmed milk or 4% BSA (MAP-2 blots), incubated with primary and secondary antibodies, and washed according to standard procedures. The values in figures are the average of the quantification of 12 blots corresponding to four different cellular pools with three independent experiments/pool.

### Immunocytochemistry

After 1 week in culture when NS were formed, some of them were immunostained using a rabbit anti-sigma-1 receptor antibody (Abcam ab53852) combined with a mouse anti-nestin antibody (Abcam ab6142). At the end of the treatment period, NS grown on glass coverslips were fixed 15–20 min at room temperature in 4% paraformaldehyde, permeabilized with 0.1% Triton X-100, and incubated at 37 °C for 1 h with the corresponding primary antibody. Then cells were washed with PBS and incubated with Alexa-488 goat anti-rabbit and Alexa-647 goat anti-mouse antibodies (1:500, Molecular Probes) for 45 min at 37 °C. To study proliferation, a rabbit anti-ki67 (1:200, Abcam ab833) antibody was used. For differentiation of NS into the different neural cell types, next antibodies were used: rabbit β-III-tubulin (1:400, TuJ-1 clone; Abcam ab68193), and mouse anti-MAP-2 (1:200, mouse; Sigma M4403) for neurons; mouse anti-GFAP (1/500, Sigma G3893) for astroglial cells, and rabbit anti-CNPase (1:100, Cell Signaling #2986) as an oligodendrocyte marker. Staining of nuclei was performed using 4′, 6-diamidino-2-phenylindole (DAPI, 1/500). Finally, images were acquired in LSM710 laser-scanning spectral confocal microscope (Zeiss). Confocal microscope settings were adjusted to produce the optimum signal-to-noise ratio. Representative images of at least eight neurospheres/condition from four different cellular pools are shown.

### Neurogenic studies in vivo

Mice housed in a 12-h light–dark cycle animal facility were divided, according to the treatment administered. (1) Short-term animals (*n* = 5 per experimental group), which received daily an intraperitoneal (i.p.) injection during 4 consecutive days with DMT (2 mg per kg of bodyweight) alone or in combination with the antagonists BD1063 (sigma-1R), methiothepin (5-HT1A/2A), ritanserin (5-HT2A) and WAY100635 (5-HT1A). On day 4, mice were intraperitoneally (i.p.) injected with 5-bromo-2-deoxyuridine (BrdU; 50 mg/kg) and sacrificed on day 5. (2) Long-term mice (*n* = 5 per experimental group), which received every other day an i.p. injection of DMT (2 mg per kg of bodyweight) alone or in combination with the correspondent antagonist during 21 consecutive days. To label proliferating cells for long-term studies on survival and differentiation, mice were intraperitoneally injected with BrdU (50 mg/kg) on day 1. All intraperitoneal treatments were administered 1 h after clorgyline (1 mg/kg, ip) injection. The dose of compounds was chosen based on previous studies^14,50,51^.

### Tissue preparation and immunohistochemistry

After treatment, the animals previously anesthetized were perfused transcardially with 4% paraformaldehyde solution, and brains were processed as previously described^52^. Sections were then incubated with anti-sigma 1 receptor rabbit (Abcam ab53852) combined with anti-nestin mouse (Abcam ab6142), anti-BrdU mouse monoclonal (DAKO M0744) combined with anti-nestin rabbit (Abcam ab7659), anti-NeuN rabbit (Millipore ABN78), or anti-doublecortin (DCX, Santa Cruz sc-8066) antibodies at 4 °C overnight, washed three times and incubated with AlexaFluor 488 goat anti-mouse and Alexa-647 goat anti-rabbit secondary antibodies for 1 h at room temperature. After rinses, sections were mounted with Vectashield. Images were obtained using a LSM710 laser-scanning spectral confocal microscope (Zeiss). Confocal microscope settings were adjusted to produce the optimum signal-to-noise ratio. Five animals from each experimental group were analyzed.

### Cell count analysis

To estimate the total numbers of cells stained with a particular marker, a modified stereological approach was used on brain coronal sections containing the SGZ, as previously described^53^. Images were processed with the image processing package Fiji^54^. Five mice per group were used. The results were expressed as the total number of labeled cells in the DG of the hippocampus by multiplying the average number of labeled cells/structure section by the total number of 30-μm-thick sections containing the DG. Values represent the means from three different experiments and five animals/experiment/experimental group. **P ≤ 0.01.

### Behavioral studies

For behavioral studies, mice were i.p. injected as mentioned above with DMT (2 mg/kg) alone or in combination with ritanserin (0.2 μg/animal) during 21 consecutive days. Then behavioral analysis was performed as previously described^45^ during 10 days, and finally, animals were sacrificed on day 31. Control animals were injected with vehicle. All intraperitoneal treatments were administered 1 h after clorgyline (1 mg/kg, ip) injection. Twelve animals per experimental group were used.

### Statistical analysis

**Fig. 1:**
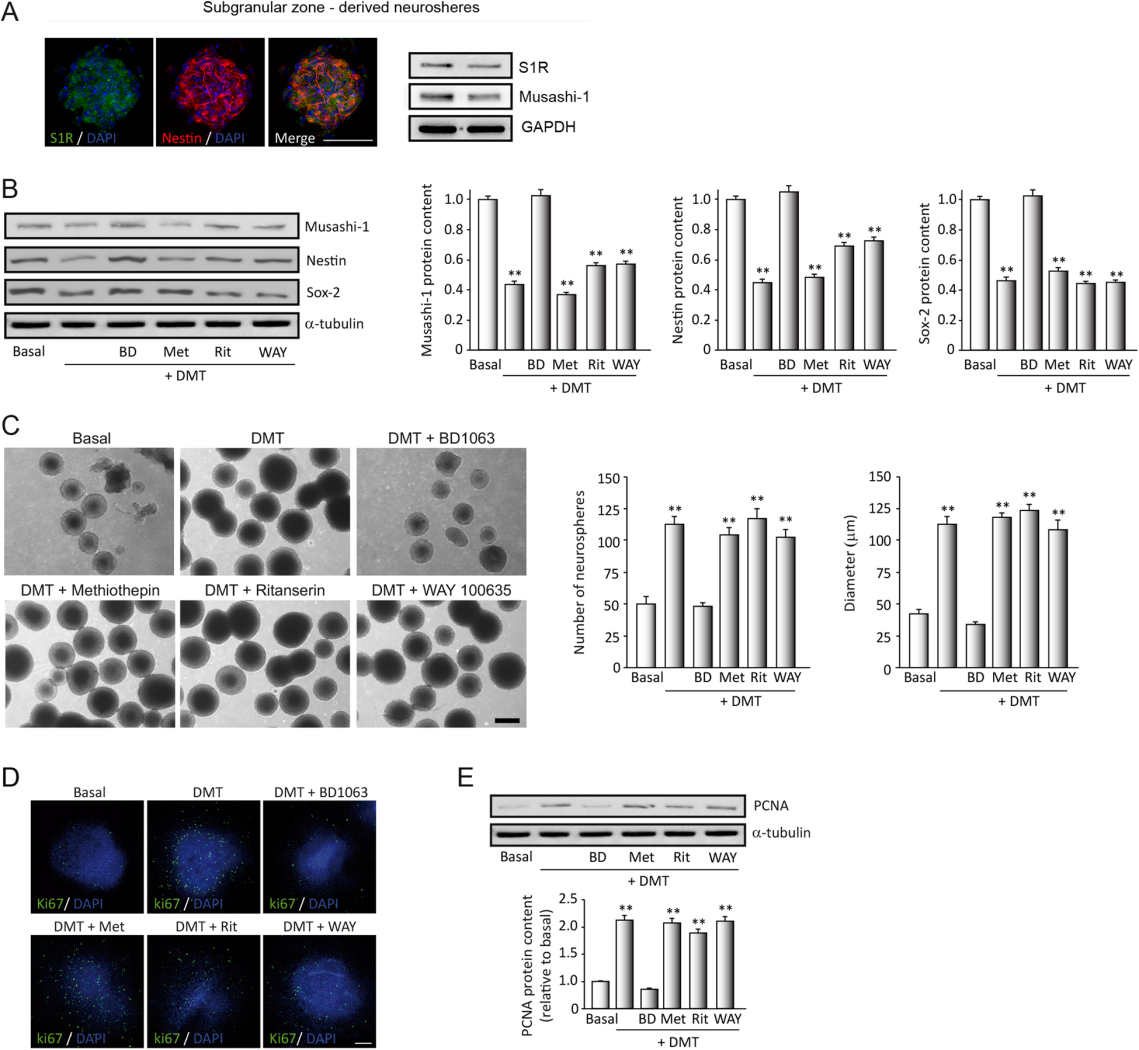
N,N-dimethyltryptamine (DMT) activates the hippocampal subgranular neurogenic niche through sigma-1 receptor (S1R). SGZ-derived neurospheres (NS) were treated during 7 days with DMT alone or in combination with the antagonists BD1063 (BD), methiothepin (Met), ritanserin (Rit), and WAY100635 (WAY). **a** Expression of the S1R (green) on neural stem cells (NSCs) (red-labeled with nestin) determined by immunocytochemistry (*n* = 8 neurospheres) and western blot analysis (*n* = 12 blots). **b** Representative western blots and quantification showing expression levels of the stemness markers musashi-1, nestin, and SOX-2 in NSCs. **c** Representative phase-contrast micrographs showing NS formation and quantification of the number and diameter of NS (*n* = 50 neurospheres per condition). Scale bar = 100 μm. **d** Confocal fluorescent images showing the expression of the cellular marker for proliferation ki67 (green) in NS after treatments (*n* = 8 per condition). DAPI was used for nuclear staining. Scale bar = 50 μm. **e** Representative western blots and quantification showing the proliferating cell nuclear antigen (PCNA) levels in NS. Values in bar graphs indicate mean ± SD of the quantification of four different cellular pools with three independent experiments/pool (*n* = 12 blots). After confirming the significance of the primary findings using ANOVA, a significance level of *P* < 0.05 was applied to all remaining post hoc (Tukey test) statistical analyses. **P ≤ 0.01 indicates significant results versus non-treated (basal) cultures.

**Fig. 2:**
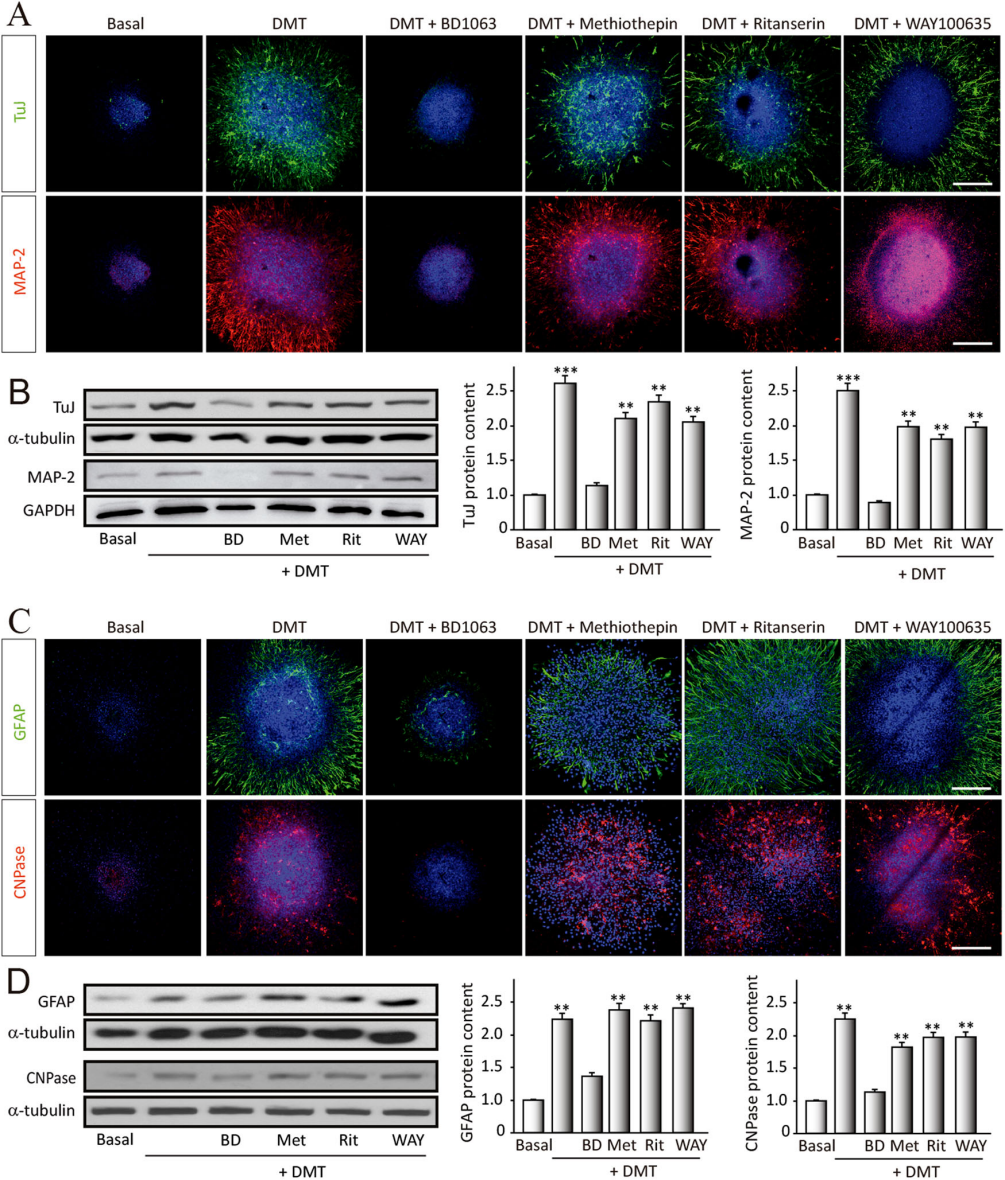
N,N-dimethyltryptamine (DMT) promotes stem cell differentiation toward all neural phenotypes. SGZ-derived neurospheres were cultured for 7 days in the presence of DMT alone or in combination with the antagonists BD1063 (BD), methiothepin (Met), ritanserin (Rit), and WAY100635 (WAY) and then were adhered on a substrate and allowed to differentiate for 3 days. **a** Confocal fluorescent images showing the expression of the neuronal markers β-III-Tubulin (TuJ-1 clone, green) and MAP-2 (red) in NS (*n* = 8 per condition). DAPI was used for nuclear staining. Scale bar = 50 μm. **b** Representative western blots and quantification of β-tubulin and MAP-2. **c** Immunofluorescence images showing NS expressing the glial fibrillary acidic protein (GFAP, red) that stains astrocytes, and in green, the oligodendrocyte marker CNPase (*n* = 8 per condition). DAPI was used for nuclear staining. Scale bar = 50 μm. **d** Representative western blots of CNPase and GFAP and quantification. Results are the mean ± SD of four different cellular pools with three independent experiments/pool (*n* = 12). After confirming the significance of the primary findings using ANOVA, a significance level of *P* < 0.05 was applied to all remaining post hoc (Tukey test) statistical analyses. **P ≤ 0.01; ***P ≤ 0.001 indicate significant results versus non-treated (basal) cultures.

**Fig. 3:**
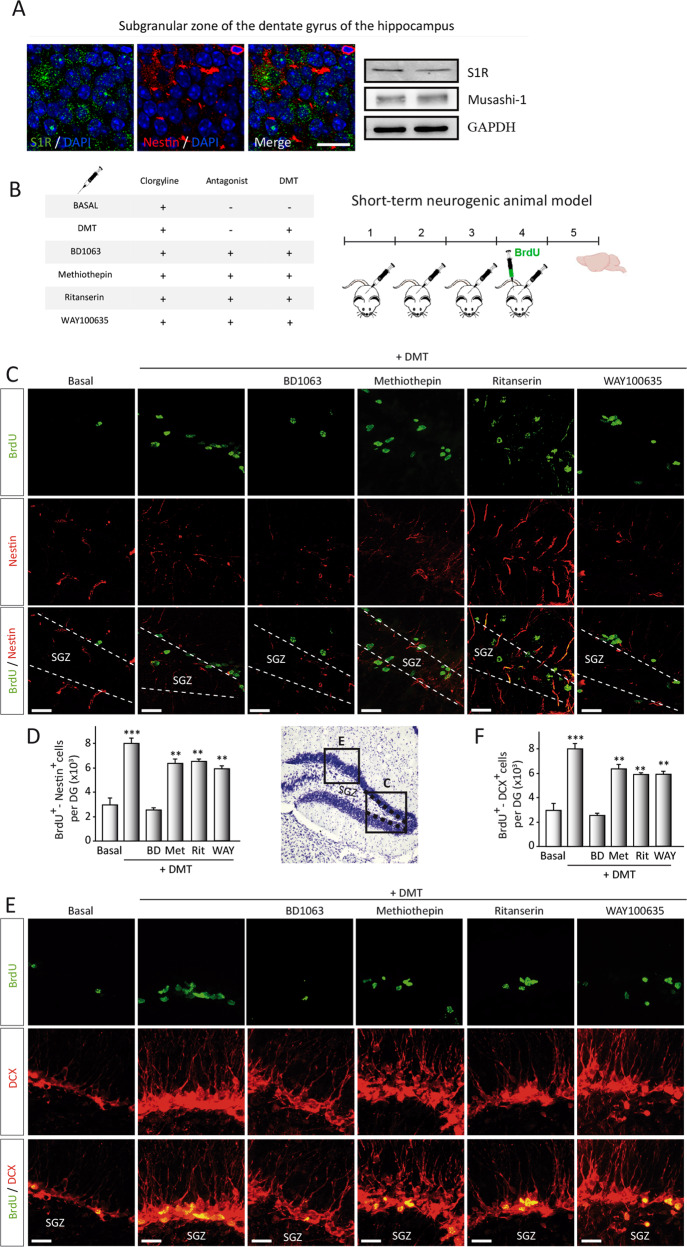
N,N-dimethyltryptamine (DMT) promotes in vivo activation of the neurogenic niche located in the SGZ of the dentate gyrus in the hippocampus. **a** Representative images (*n* = 3 animals) showing the expression of the sigma-1 receptor (S1R, green) on neural stem cells (NSCs) (red-labeled with nestin) in the subgranular zone of the dentate gyrus. S1R expression was also determined by western blot on hippocampal tissue (*n* = 3 animals). **b** Schematic representation of experimental design and treatment schedule for in vivo short-term neurogenic study. DMT alone or in combination with the antagonists BD1063 (BD), methiothepin (Met), ritanserin (Rit), or WAY100635 (WAY) was daily intraperitoneally injected during 4 days. To label proliferating cells, on day 4 mice were i.p. injected with 5-bromo-2-deoxyuridine (BrdU) and sacrificed on day 5. **c** Confocal maximum intensity projection images showing BrdU/Nestin co-staining on the SGZ of adult mice. BrdU is shown in green and nestin in red. Scale bar = 25 μm. **d** Quantification of the number of double BrdU/Nestin cells in the DG performed on confocal orthogonal images is shown. **e** Double BrdU^+^-DCX^+^ expressing cells in the SGZ. Confocal maximum intensity projection images show BrdU in green and DCX in red. Scale bar = 25 μm. **f** Quantification of the number of BrdU^+^- DCX^+^ cells in the DG is shown. All quantification values represent the mean ± SD (*n* = 5 animals per group). After confirming the significance of the primary findings using ANOVA, a significance level of *P* < 0.05 was applied to all remaining post hoc (Tukey test) statistical analyses. **P ≤ 0.01; ***P ≤ 0.001 indicate significant results versus vehicle-treated (basal) animals.

**Fig. 4:**
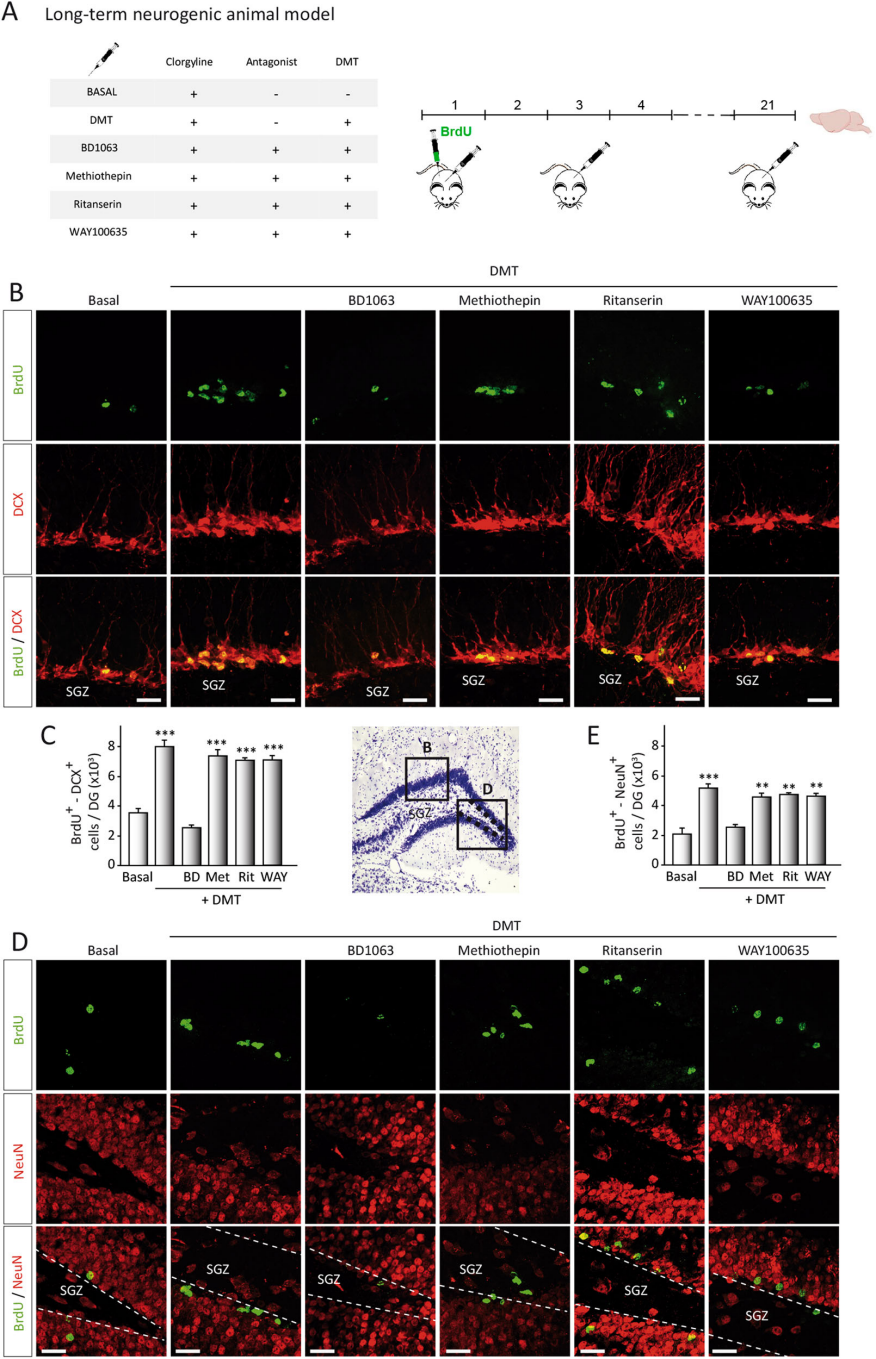
N,N-dimethyltryptamine (DMT) promotes in vivo neurogenesis on the subgranular zone of the dentate gyrus in the hippocampus. **a** Schematic representation of the experimental design and treatment schedule for in vivo long-term neurogenic study. DMT alone or in combination with the antagonists BD1063 (BD), methiothepin (Met), ritanserin (Rit), or WAY100635 (WAY) was intraperitoneal injected on alternate days during 21 days. To label proliferating cells, on day 1 mice were i.p. injected with 5-bromo-2-deoxyuridine (BrdU) and sacrificed on day 21. **b** BrdU-DCX-expressing cells in the DG. Representative confocal images are shown. Scale bar = 25 μm. **c** Quantification of the number of BrdU^+^-DCX^+^-expressing cells in the DG based on confocal orthogonal images. **d** Confocal maximum intensity projection images showing the colocalization of BrdU (green) and neuN (red) cells in the dentate gyrus of the hippocampus of adult mice. Scale bar = 25 μm. **e** Quantification of the number of BrdU^+^/NeuN^+^ cells performed on confocal orthogonal images in DG. Values represent the mean ± SD (*n* = 5 animals per group). After confirming the significance of the primary findings using ANOVA, a significance level of *P* < 0.05 was applied to all remaining post hoc (Tukey test) statistical analyses. **P ≤ 0.01; ***P ≤ 0.001 indicate significant results versus vehicle-treated (basal) animals.

**Fig. 5:**
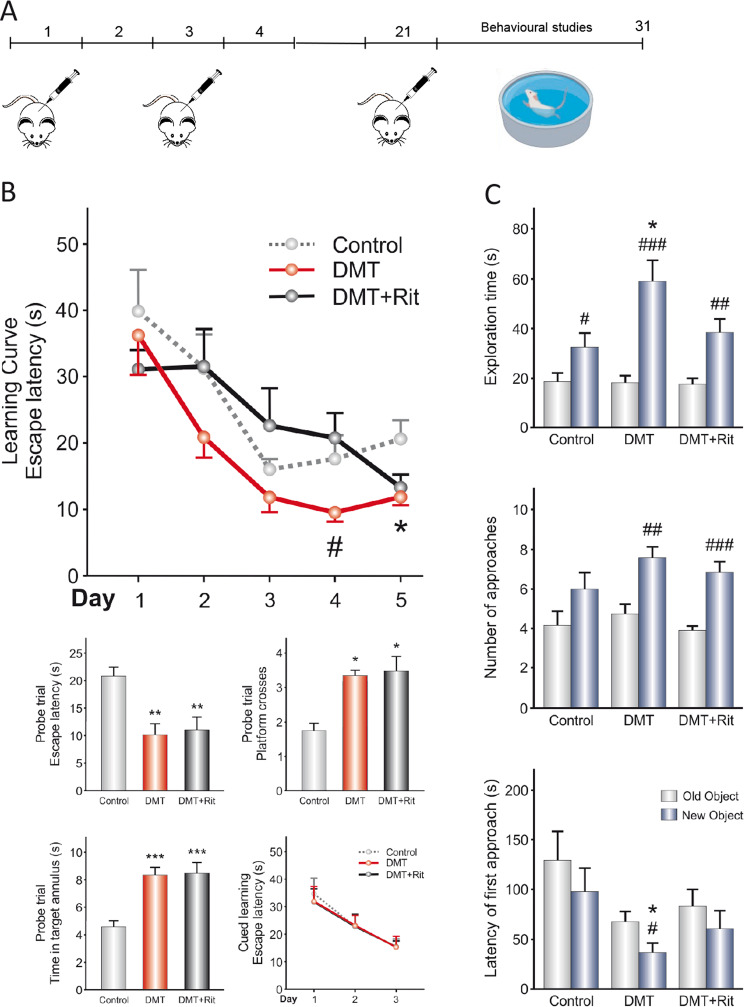
N,N-dimethyltryptamine (DMT) promotes improved performance in learning tasks linked to hippocampal neurogenesis. **a** Schematic representation of the experimental design for behavioral tests. DMT alone or in combination with the antagonist ritanserin (Rit) was intraperitoneally injected on alternating days for 21 days. Then, behavioral tests were performed for 10 days, and finally animals were sacrificed on day 31. **b** Data from Morris water maze test. *P < 0.05; **P < 0.01; ***P < 0.001 versus the control group. ^#^*P* < 0.05 versus DMT + ritanserin group. **c** Data from the novel object recognition test. Values represent the mean ± SEM (*n* = 12 per group). *P < 0.05 versus the control group. ^#^*P* < 0.05; ^##^*P* < 0.01; ^###^*P* < 0.001 versus old object. After confirming the significance of the primary findings using ANOVA, a significance level of *P* < 0.05 was applied to all remaining post hoc (Tukey test) statistical analyses.

No statistical methods were used to determine the sample size for each experiment. The sample size and animal numbers were estimated based on our previous studies. For animal studies, no randomization was used. Statistics analysis data from Figs. 1–5 were analyzed using a one-way ANOVA. In Fig. 5, data from the learning curve and cued learning were analyzed using a two-way mixed ANOVA. After confirming the significance of the primary findings using ANOVA, a significance level of *P* < 0.05 was applied to all remaining post hoc statistical analyses (Tukey test). The SPSS statistical software package (version 20.0) for Windows (Chicago, IL) was used for all statistical analyses.

## Results

### DMT controls the stemness of neural progenitors in vitro through the S1R

We first analyzed whether the sigma-1 receptor (S1R) was expressed on murine NSCs isolated from the subgranular zone of the dentate gyrus of the hippocampus. Figure 1a shows S1R expression on neurospheres in the basal state determined by immunocytochemistry and western blot analysis. To analyze the “stemness” of cultured neurospheres, we determined the expression of potentiality markers of this state. Then, we performed WB analysis after treatment of these cultures during 7 days under proliferative conditions (see “Materials and methods”) with DMT alone or in combination with the different antagonists. Our results (Fig. 1b) show significant reductions in protein levels of musashi-1, nestin, and SOX-2 in the SGZ-derived neurospheres after treatment with DMT, suggesting a loss of stemness in NSCs in the NS cultures. When these cultures were pre-treated with BD1063, a specific antagonist for S1R, this effect was reversed, and stemness marker levels were similar to those observed in basal conditions. On the contrary, the expression of stemness markers in those cultures treated with DMT together with the mixed serotonin 5-HT1A/2A receptor antagonist methiothepin, the selective 5-HT2A receptor antagonist ritanserin, or the selective 5-HT1A receptor antagonist WAY100635, significantly decreased stemness as occurred in DMT-treated cultures. These results suggest that DMT promotes a loss of “stemness” or and undifferentiated state of the neurospheres, through the S1R.

### DMT promotes the proliferation in vitro of NSCs

Other NS cultures were used to study proliferation; thereby, the number and diameter of the neurospheres were evaluated (Fig. 1c). DMT notably increased the number and size of the neurospheres in NS cultures after 7 days of treatment, indicating that DMT promotes the proliferation of adult hippocampal-derived neural progenitors. DMT proliferative effect was blocked when cultures were treated with BD1063 showing a significant decrease in the number and size of neurospheres, similar to basal conditions. Also, significant differences, in number and size of neurospheres, were observed when cultures were treated with DMT combined with methiothepin, ritanserin, or WAY100635.

We next analyzed changes in two well-known markers for proliferation, ki67 and proliferating cell nuclear antigen (PCNA) (Fig. 1d, e). Fluorescent immunocytochemical analysis of ki67 expression (Fig. 1d) showed an increase in the number of ki67^+^ cells in the NS after treatment with DMT, suggesting a direct effect of DMT on the proliferation ability of NSCs. This effect was clearly reverted when cultures were also incubated with the antagonist BD1063 (BD). Similar results were obtained by western blot analysis and subsequent quantification of PCNA (Fig. 1e). No significant differences in the expression of ki67 and PCNA were observed when cultures were preincubated with other DMT antagonists. These results indicate that DMT stimulates in vitro, through the S1R, the proliferation of neural progenitors of the adult neurogenic niche of the hippocampus.

### DMT promotes the differentiation in vitro of NSCs toward the three main neural cellular types

Treated neurospheres during 7 days in the presence of DMT, alone or in combination with the different antagonists, under differentiation conditions (medium with 1% fetal bovine serum and absence of growth factors) were used. To study the ability to differentiate into a certain neural phenotype, the expression of specific proteins linked to every neural subtype was analyzed (Fig. 2). To detect neurons, β-III-tubulin (clone TuJ-1), found exclusively in neurons and MAP-2 (microtubule-associated protein 2), present in mature neurons, were used (Fig. 2a, b). To study its differentiation toward an astroglial or oligodendroglial phenotype (Fig. 2c, d), we analyzed the expression of GFAP (astrocytes) and CNPase (oligodendrocytes).

Figure 2a, b shows a striking increase in the expression of β-III-tubulin and MAP-2 in neurospheres treated with DMT, compared with basal (non-treated) cultures. This neurogenic effect is clearly blocked by BD. No differences in the expression of neuronal markers were observed when cultures were treated with DMT in combination with metiotepine, ritanserin, or WAY. These results suggest that DMT stimulates the in vitro differentiation of neural progenitors toward a neuronal phenotype through S1R.

Related to gliogenesis, Fig. 2c, d shows an increase in the expression levels of GFAP and CNPase, after DMT treatment. This promotion of astroglial cells and oligodendrocytes generation was blocked when cultures were pre-treated with the antagonist BD. We did not observe differences in the expression of GFAP and CNPase when neurospheres were pre-treated with the other antagonists. These results may suggest a direct effect of DMT on in vitro differentiation of neural progenitors toward astrocytes and oligodendrocytes via S1R.

### DMT activates in vivo the subgranular zone neurogenic niche in adult mice

To confirm our in vitro results on the role of the S1R on DMT neurogenic action, we first determined the expression of S1R in the subgranular neurogenic niche. To that end, brain coronal sections, including the hippocampus and protein samples isolated from the SGZ, were analyzed. As can be observed in Fig. 3a, immunofluorescence on the subgranular zone and western blot analysis shows the expression of S1R in this brain area. We next analyzed whether DMT also exerted an effect stimulating the proliferation kinetics of NSCs in the SGZ in vivo. To that end, adult mice were intraperitoneally injected during 4 (short-term) or 21 days (long-term) with DMT alone or in combination with antagonists, followed by BrdU administration for 24 h (Fig. 3) or 21 days (Fig. 4) before sacrifice. In short-term animals (Fig. 3b), immunohistochemical and cell count analysis performed on brain serial coronal sections containing the SGZ (Fig. 3c, d) demonstrated that DMT significantly increased the number of double BrdU/Nestin-stained cells in the SGZ, in comparison with control values (clorgyline-treated group). This neurogenic stimulation seemed to be mediated by the S1R since no neurogenic effect was observed when DMT is administered together with the antagonist BD1063. No differences were found in BrdU and nestin immunostaining in those animals injected with DMT in combination with the antagonist methiothepin and WAY100635.

During the neurogenic process, proliferation is crucial but also the migration of the newly generated precursor from the SGZ to the granular layer. To study the migration of neural precursors, serial coronal brain sections were stained for doublecortin (DCX). The results shown in Fig. 3e, f show a higher immunopositive BrdU/DCX cells in the SGZ of DMT-treated animals. Additionally, DCX-stained cells in DMT-treated animals exhibited extensive dendritic arborizations. No difference was observed when animals were treated with DMT combined with antagonists. Contrarily, when DMT was injected with BD1063, Brdu, or DCX expression was not increased. These results confirm that DMT-treated mice exhibit enhanced proliferation and migration of neural precursors in the SGZ after 4 days of treatment, suggesting a modulating effect of this compound on hippocampal neurogenesis in vivo.

In order to know whether these new migrating neuroblasts were able to properly reach the granular cell layer, long-term (21 days) treated animals were used (Fig. 4a). Quantification analysis of confocal images demonstrates an increase in DCX^+^/BrdU^+^-cell in the SGZ after DMT treatment (Fig. 4b, c). No differences were found in those animals treated with DMT together with methiothepin or WAY100635. Once again, combined treatment of DMT with BD1063 blocked the migration increase observed in animals treated only with DMT. In addition, at this time when neuroblasts have reached the granular cell layer, a noticeable increase in the amount of newly generated neurons (BrdU^+^/NeuN^+^ cells) was seen in this layer (Fig. 4d, e) in DMT-treated animals. This increase in the number of newly generated granular cells was blocked when mice treated with DMT together with BD1063. Altogether, these observations clearly indicate that DMT increases in vivo the number of new neurons originated in the hippocampus, action mediated by S1R.

Taking into account these results, we finally analyzed the functional consequences of DMT treatment by performing behavioral tasks (Fig. 5a) to analyze if memory and learning are affected. Figure 5b (left panel) shows the results obtained by the Morris water maze test. During the learning curve, there were significant differences between groups only on days 4 and 5, showing that the DMT group tended to reduce the escape latency compared with DMT + ritanserin and control groups, respectively. In the probe trial, DMT and DMT + ritanserin groups showed a significant reduction in escape latency compared with the control group. In this line, we found that the control group performed less platform crosses and spent less time in target annulus around the previous platform location. Data from the probe trial indicate that the DMT and DMT + ritanserin remembered more effectively the zone where the hidden escape platform was. During the 3 days of cued learning, no differences were observed between groups, indicating that the differences observed in the learning curve and probe trial were not due to differences in the motivation of animals to escape from the water nor sensorimotor abilities.

Regarding the new object recognition test (Fig. 5c, right panel), the DMT group showed a longer exploration time of the new object and a greater number of approaches to it. In addition, this group tended to explore the new object before the old one. DMT + ritanserin group spent more time exploring the new object and approached it more times. Finally, the control group only spent more time exploring the new object. In addition, we obtained differences in the latency of the first approach and exploration time between DMT and control groups. These results suggest that DMT and DMT + ritanserin groups showed better episodic memory compared to the control group.

## Discussion

We have previously described that β-carbolines alkaloids, the three main alkaloids present in *Banisteriopsis caapi* and harmol, the main metabolite of harmine in humans, play an important role as key regulators on adult neural stem cell activity^49^. Using an in vitro model of adult neurogenesis, we showed that they promote the proliferation and migration of progenitor cells and induced their differentiation mainly into a neuronal phenotype. The main limitation to that work was that the potential role of DMT, other active compounds contained in ayahuasca brews, was not described. Moreover, previous studies performed on rodents and primates^55–58^, and more interestingly in humans^4,59^, suggest that ayahuasca infusion has antidepressant activity, a therapeutic effect usually linked to hippocampal neurogenesis. This work extends our previous results indicating the role of DMT, one of the main compounds of the hallucinogenic infusion ayahuasca, in adult neurogenesis.

Our results in vitro and in vivo show that DMT is a key regulator in the activity of adult NSCs, since this compound plays an important role in regulating the expansion and differentiation of the stem cell population located in the SGZ, one of the main adult neurogenic niches. This is revealed in vitro by an increase in the number and size of primary neurospheres and an increased expression of ki67 and PCNA, which indicates a high rate of proliferation and loss of stemness after treatment with DMT. Increased proliferation does not indicate neuronal commitment^60^; however, DMT also induced an increase in β-III-tubulin^+^ and MAP-2^ +^ cells, suggesting promotion of differentiation toward a neuronal phenotype and increasing the total numbers of the neuron that reach neuronal maturity. Interestingly, in contrast to that previously described on the action of carbolines in vitro^49^, we have also found an increase in the number of other neural cells such as astrocytes and oligodendrocytes after DMT treatment. Similar results were observed in vivo, with an increased proliferation rate of the NSCs and a larger population of doublecortin expressing neuroblasts migrating to the hippocampal granular layer to generate new neurons. Moreover, these have a functional impact since DMT treatment during 21 days clearly improved mouse performance in learning and memory tasks, in which the hippocampus is considered to play an essential role. These observations are in agreement with previous works showing that adult hippocampal neurogenesis plays an important role in these cognitive functions^61–65^. Considering these effects, we can determine that the DMT has the capacity to regulate the expansion and destination of stem cell populations and therefore contribute to memory and learning processing in the dentate gyrus.

Neurogenesis consists of proliferation and loss of stemness of the NSCs, migration of neuroblasts and differentiation into functional neurons. Results here obtained demonstrate that DMT controls all these stages. Interestingly, in addition to the neurogenic potential, DMT also induced the formation of astrocytes and oligodendrocytes. This ability for controlling neurogenesis is of great interest, since in pathological conditions, the renewal of the neurons must be optimized by acting simultaneously on several processes^40,66^. We have previously indicated that many molecules^67–71^ and recently β-carbolines contained in ayahuasca^49^ exerted and effect on cell proliferation and differentiation, therefore the effect of DMT stimulating cell proliferation and differentiation is not exclusive to this compound. One of the goals of this work is that additionally to its neurogenic effect, DMT also stimulated migration and new generation of astroglial cells and oligodendrocytes, what highlights the versatility of this compound as it can promote all the processes involved in full adult neurogenesis. Specifically, astrocytes are known to support the proliferation, survival, and maturation of developing neurons and neuroblasts that have already committed to neuronal lineages^72^ but also to promote neurogenesis^73,74^. In fact, previous works demonstrated that astrocytes in vitro could be directly converted into neurons or stem-like cells, pointing to the plasticity of these somatic glial cells^75–77^. No previous studies on the neurogenic effect of DMT have been described, but in comparison with the effect of other ayahuasca components such as β-carbolines^49^, we can conclude that the effect of DMT on adult neurogenesis is considerably more potent. As an additional value to the generation of neurons, the glial cells formation induced by DMT might be an ideal target for in vivo neuronal conversion after neural injury, since some studies have achieved to generate proliferating, non-tumorigenic neuroblasts from resident astrocytes^78^. The main therapeutical implication of the results here obtained is derived from the close relationship between neurogenesis and antidepressant activity described in several animal models^79^.

DMT is considered a serotoninergic drug because its mechanism of action consists in agonism at different serotoninergic receptors, especially the 5-HT2A receptors widely described as inducers of neurogenesis^80^, but also psychedelics (reviewed by Dos Santos and Hallak^81^). One of the main limitations that arise when designing a possible drug from the results obtained is to achieve the desired neurogenic effect without causing the patient hallucinogenic effects secondary to treatment with DMT, through the activation of 5-HT2A receptors. The results here obtained indicate that the observed effects of DMT are mediated by the activation of the S1R. In this regard, it has been shown that the stimulation of the S1R by different agonists enhances neurogenesis in the hippocampus^23,82^. Moreover, in vivo evidence suggests that the σ1R deficiency interrupts the adult neurogenesis^22^. In humans, the use of S1R agonists, such as fluvoxamine, shows its involvement in neuroplasticity^83^, suggesting an important role in improving learning mechanism. In clinical studies, some S1R agonists, including fluvoxamine, donepezil, and neurosteroids, improve cognitive impairment^84,85^. Adult hippocampal neurogenesis is widespread in mammals, including humans, and may act as a key regulator in cognition, memory, and emotion-related behavior^86^. Deficits in adult neurogenesis are associated with the physiopathology of depression and modulation of neurogenesis is behind the action of several antidepressants^79^.

Recently, a previous study has described the role of other psychoactive tryptamine, 5-methoxy-N,N-dimethyltryptamine (5-MeO-DMT), in neurogenesis^87^. In contrast to the intracerebral injection of 5-MeO-DMT administered by these authors, we used DMT i.p. that can cross the blood–brain–barrier, which facilitates its future administration in humans. Moreover, the neurogenic effect of DMT through the S1R activation is combined to the antagonism of 5-HT2A receptor, avoiding the hallucinogenic effects of these tryptamine derivatives. This information could be very useful for the future development of new treatments against neurodegeneration.

In conclusion, this study shows that DMT present in the ayahuasca infusion promotes neurogenesis by stimulating the expansion of neural progenitors populations, and by inducing the differentiation of these NSCs. Moreover, the neurogenic stimulation observed after DMT treatment correlates with an improvement in spatial learning and memory tasks in vivo. Stimulation of the neurogenic niches of the adult brain can contribute substantially to the antidepressant effects of ayahuasca in recent clinical studies. The versatility and complete neurogenic capacity of the DMT guarantee future research regarding this compound. In addition, its ability to modulate brain plasticity indicates its therapeutic potential for a wide range of psychiatric and neurological disorders, among which are neurodegenerative diseases.

## Supplementary information

Supplemental material
