# Supplementary material for: N,N-dimethyltryptamine compound found in the hallucinogenic tea ayahuasca, regulates adult neurogenesis in vitro and in vivo
Source: Transl Psychiatry. 2020 Sep 28;10:331. doi: 10.1038/s41398-020-01011-0 (PMC7522265; doi:10.1038/s41398-020-01011-0)
Supplement: Supplementary file 1 — Supplemental material [file 41398_2020_1011_MOESM1_ESM.docx]

Supplemetary Information.

*Drugs*

N,N-dimethyltryptamine (DMT) was acquired from Dr. Carmen Escolano from the laboratory of Medicinal Chemistry at Faculty of Pharmacy and Food Sciences, and Institute of Biomedicine (IBUB), University of Barcelona, Spain. The selective Sigma-1 receptor antagonist BD1063 (BD, Catalog number SML0276), the mixed serotonin 5-HT1A/2A receptor antagonist methiothepin (Met, Catalog number M149) and the selective 5-HT2A receptor antagonist ritanserin (Rit, Catalog number R103) were purchased from Sigma-Aldrich. Selective 5-HT1A receptor antagonist WAY100635 (WAY, Catalog number ab120550) was acquired from Abcam and clorgyline hydrochloride (Catalog number MM3019.00) was obtained from LGC Standards.

*Western blot antibodies.*

Primary antibodies used were: sigma 1 receptor (1/1000, rabbit; Abcam ab53852), musashi-1 (1/500, rabbit; Abcam ab21628), nestin (1/500, rabbit; Abcam ab7659), SOX-2 (1/1000, mouse, Cell Signaling L1D6A2), PCNA (1/500, mouse, Millipore MABE288), β-III-tubulin (1/1000, TuJ clone; mouse; Covance MMS-435P) MAP-2 (1/500, mouse; Sigma M4403), CNPase (1/500, rabbit, Cell Signaling #2986) and GFAP (1/1000, mouse; Sigma G3893). For each sample, the α-tubulin (1/5000, mouse; Sigma T9026) or GAPDH (1/5000, mouse, Millipore MAB374) level expression was determined as a loading control. Secondary peroxidase-conjugated (1/1000) donkey anti-rabbit (GE Healthcare NA934), or rabbit anti-mouse antibodies (Jackson Immunoresearch 315-035-008) were used.

*Cell Count analysis*

BrdU/Nestin and BrdU/NeuN coexpression was defined by nuclear colocalization of the two markers over the extent of the nucleus in consecutive 0.5 mm z-stacks, when green (BrdU) and red (Nestin, DCX or NeuN) signals coincided, and when colocalization was confirmed in x-y, x-z and y-z cross-sections produced by orthogonal reconstructions from z-series. Only contrast enhancements and color level adjustments were made; otherwise images were not digitally manipulated. From serial coronal sections (30 μm) from the entire rostrocaudal extent of the DG, every sixth section was selected to count the number of immunoreactive cells for a given marker. The boundaries of these nervous system regions were determined with reference to internal anatomic landmarks ^1^. For each area of interest, images were analyzed using computer-assisted image analysis software (Soft Imaging System Corporation). Positive cells, which intersected the uppermost focal plane (exclusion plane) and the lateral exclusion boundaries of the counting frames, were not counted.

*Behavioral studies*

Morris Water Maze

The Morris Water Maze (MWM) was used to measure spatial learning and memory. The test was carried out during 9 consecutive days in a circular pool (diameter 122 cm) filled with colored water (temperature 23 ± 1ºC). The pool was located in a room with visible external cues and light intensity controlled. A hidden escape platform (diameter 10 cm) was submerged 0.5-1 cm below the water surface in one of four equal imaginary quadrants. The learning trials were conducted over 5 days, with 4 trials each day, with an interval between trials of 5 minutes. Mice were daily trained to find the submerged platform and escape from water by swimming from a semi-random set of start positions. The animals had a limit of 60 seconds per trial to find the platform. Escape latencies were recorded in each trial and the average was calculated for each day. After each trial, mice were dried and returned to their home cages. To analyze reference memory at the end of learning, one probe trial without platform was carried out for 60 seconds with a new start position in the pool to ensure that the mice remember the goal location rather than a specific swim path. This trial was carried out 24 h after the last learning day. The analyzed variables during the probe trial were the latency to reach the previous platform location, the number of platform-site crossovers and the time spent within an imaginary ring (diameter 30 cm) around the previous platform location. All sessions were recorded by a video camera located above the pool and an experimenter blind to the treatments analyzed the videos. After the spatial version of the MWM, we tested the motivation of animals to escape from the water and the sensorimotor abilities in a cued learning. Mice were trained to find the submerged platform indicated by a visible ‘flag’. All animals received 4 trials over three consecutive days in which the cued platform and the start position were moved to a new location on every trial. Escape latencies were recorded in each trial and the average was calculated for each day ^2^.

Novel Object Recognition Test

Novel object recognition (NOR) test was conducted to assess the episodic memory. The test was carried out using six 25 x 25 cm boxes. Two sets of objects were used during this test, one set of objects was made of black plastic (12-cm high and 4.5-cm wide) and another set of objects was made of clear blue plastic (11-cm high and 5-cm wide). NOR test was carried out for two days following the protocol described in Bevins & Besheer, ^3^. On day 1 (habituation session), each mouse was allowed to habituate during 10 minutes to the test box. On day 2 mice were placed in the box and allowed to explore two identical sample objects for 10 minutes (familiarization session). Following, the mice were returned to their cages for 1 hour (retention interval). Then, mice were placed in the same box with one familiar and one novel object (counterbalanced across mice) and given 5 min to explore them (test session). All the sessions were monitored by a video camera above the apparatus. An experimenter blind to the treatments measured the time the animals spent exploring each object, the latency of the first approach and the number of approaches to each object. An object approach was any contact with the mouth, nose or paw. Accidental contacts such as backing into the object or bumping the object as it passes were not included as approaches.

1. Paxinos G, Franklin KBJ. *The mouse brain in stereotaxic coordinates*, 2019.

2. Vorhees CV, Williams MT. Morris water maze: procedures for assessing spatial and related forms of learning and memory. *Nat Protoc* 2006; **1**(2)**:** 848-858.

3. Bevins RA, Besheer J. Object recognition in rats and mice: a one-trial non-matching-to-sample learning task to study 'recognition memory'. *Nat Protoc* 2006; **1**(3)**:** 1306-1311.
